# Right ventricular stroke volume assessed by pulmonary artery pulse contour analysis

**DOI:** 10.1186/s40635-020-00347-7

**Published:** 2020-10-07

**Authors:** David Berger, Jan Hobi, Per W. Möller, Matthias Haenggi, Jukka Takala, Stephan M. Jakob

**Affiliations:** 1 Department of Intensive Care Medicine, Inselspital, Bern University Hospital, University of Bern, CH-3010 Bern, Switzerland; 2 Department of Anaesthesiology, Alingsas Hospital, Alingsås, Sweden

**Keywords:** Pulse contour analysis, Right ventricle, Stroke volume

## Abstract

**Background:**

Stroke volume measurement should provide estimates of acute treatment responses. The current pulse contour method estimates left ventricle stroke volume. Heart-lung interactions change right ventricular stroke volume acutely. We investigated the accuracy, precision, and trending abilities of four calibrated stroke volume estimates based on pulmonary artery pulse contour analysis.

**Results:**

Stroke volume was measured in 9 pigs with a pulmonary artery ultrasound flow probe at 5 and 10 cmH_2_O of PEEP and three volume states (baseline, bleeding, and retransfusion) and compared against stroke volume estimates of four calibrated pulmonary pulse contour algorithms based on pulse pressure or pressure integration. Bland-Altman comparison with correction for multiple measurements and trend analysis were performed. Heart rate and stroke volumes were 104 ± 24 bpm and 30 ± 12 mL, respectively. The stroke volume estimates had a minimal bias: − 0.11 mL (95% CI − 0.55 to 0.33) to 0.32 mL (95% CI − 0.06 to 0.70). The limits of agreement were − 8.0 to 7.8 mL for calibrated pulse pressure to − 10.4 to 11.5 mL for time corrected pressure integration, resulting in a percentage error of 36 to 37%. The calibrated pulse pressure method performed best. Changes in stroke volume were trended very well (concordance rates 73–100%, *r*^2^ 0.26 to 0.987, for pulse pressure methods and 71–100%, *r*^2^ 0.236 to 0.977, for integration methods).

**Conclusions:**

Pulmonary artery pulse contour methods reliably detect acute changes in stroke volume with good accuracy and moderate precision and accurately trend short-term changes in cardiac output over time.

## Introduction

The monitoring of cardiac output or stroke volume as a cornerstone of perioperative and critical care is recommended for high-risk surgical patients [1] and patients in persistent shock [2]. Thermodilution with the pulmonary artery catheter is considered the clinical reference method. Stroke volume variation (SVV) based on calibrated or uncalibrated arterial pulse contour analyses [3] is commonly used to assess volume responsiveness or preload dependency (which does not equal the need for volume [4, 5]). Increased right ventricular afterload and right ventricular dysfunction may also result in increased SVV and misleadingly suggest volume responsiveness in pulmonary hypertension [6], right heart failure or pulmonary parenchymal diseases [7, 8], and changing left ventricular afterload conditions [9]. In such clinical situations, the use of a pulmonary artery catheter (PAC) facilitates the assessment of right ventricular stroke volume and differentiation of stroke volume variation caused by right ventricular failure or volume dependency. Despite the lack of proven outcome benefits from its use, the PAC provides the unique ability to assess the right heart and pulmonary circulation and to integrate pulmonary gas exchange into hemodynamic assessment [10, 11]. In contrast to pulse contour methods, current thermodilution methods are not feasible for detecting rapid changes in cardiac output due to slow response time [12] and limited repetitions of intermittent boli. A fast-responding cardiac output measurement is necessary for the dynamic assessment of heart-lung interactions and fluid responsiveness with stroke volume variation [5]. Pulmonary artery (PA) pulse contour analysis could provide a method for rapid assessment of changes in right ventricular stroke volume. The right ventricular output is sensitive to changes in afterload and venous return, whereas the elastic properties of the pulmonary circulation may clearly differ from the systemic arterial tree. The specific behavior of pulmonary vascular impedance characteristics [13, 14] may necessitate adapted calculation models. Pulmonary artery pulse contour analysis was investigated in the 1970s [15–18], but not pursued further despite promising results. The reliable method of intermittent PAC thermodilution used in combination with a calibrated PA pulse contour analysis would provide both a reference cardiac output monitor and a fast-response measure of right-sided stroke volume suitable for dynamic assessment of heart-lung-interaction. The aim of this study was to investigate the precision, accuracy, and trending abilities of four simple pulmonary artery pulse contour methods with Bland-Altman analysis and their interdependence on changes in pulmonary artery elastance and changing volume state.

## Methods

This analysis was an independent, post hoc sub-study of an experiment on respiratory maneuvers and venous return. Baseline characteristics have been published before [19, 20]. The study was conducted in accordance with the Guide for the Care and Use of Laboratory Animals (National Academy of Sciences, 1996) and Swiss National Guidelines and was approved by the Commission of Animal Experimentation of Canton Bern, Switzerland (approval BE 71/14), and this manuscript adheres to the applicable ARRIVE guidelines. Additional details on anesthesia and the surgical preparation can be found in reference [19]. In brief, 10 pigs (39.1 ± 1.7 kg body weight) were equipped with a transit time ultrasonic flow probe (PAU Series, Transonic Systems, Ithaca, USA) on the pulmonary artery via a sternotomy. Pulmonary artery pressure (PAP) was measured via a catheter surgically inserted into the pulmonary artery (PA). The measurements were taken in closed-chest conditions without suction on the pleural drains. After surgery, a stabilization period followed, in which a bolus of 100 mL hydroxyethylstarch was given, followed by one repeat 100 mL bolus if stroke volume increased > 10%. This is referred to as *baseline* [19].

The experimental protocol consisted of measurements at *PEEP 5 cmH*_*2*_*O* (volume state like in baseline) and *10 cmH*_*2*_*O* in order to investigate the effects of increased intrathoracic pressures with increased afterload on the stroke volume estimates. In the second step, stroke volume was altered from *baseline* (at stable PEEP 5 cmH_2_O) by stepwise *bleeding* (6 and 3 mL/kg body weight) and *retransfusion* with volume expansion of the shed blood diluted with 1:1 hydroxyethyl starch [19].

Data was recorded in Labview (National Instruments, TX, USA) with a sampling rate of 100 Hz and extracted with custom-made software (Soleasy, Alea Solutions, Zürich, Switzerland).

### Tested methods

We tested four, previously published, calibrated pulse contour algorithms for stroke volume determination from the pulmonary artery pressure tracing, identified in a literature review. The stroke volume estimations were based on pressure amplitude or time-pressure curve integration:

1. Calibrated pulse pressure:

SV = *K*_1_ × PP [15]

2. Calibrated pulse pressure with correction for systole time:

SV = *K*_2_ × PP × *T*_*s*_ [15, 21]

3. Pressure integral:

$$ \mathrm{SV}={K}_3\times \underset{T_0}{\overset{T_e}{\int }}\left({P}_{\mathrm{PA}}-{P}_{\mathrm{ed}}\right) dt $$ [16, 22]

4. Pressure integral with time correction:

$$ \mathrm{SV}={K}_4\times \underset{T_0}{\overset{T_e}{\int }}\left({P}_{\mathrm{PA}}-{P}_{\mathrm{ed}}\right) dt\left(1+\raisebox{1ex}{${T}_s$}\!\left/ \!\raisebox{-1ex}{${T}_D$}\right.\right) $$ [17, 18]

where *K*_*x*_ is a calibration constant for the respective method; PP indicates pulse pressure (or amplitude); *P*_PA_ and *P*_ed_ indicate the instantaneous pulmonary artery and diastolic pressure, respectively; *T*_e_ is the time point of end-systole (as defined by the occurrence of the dicrotic notch); *T*_*s*_ and *T*_*d*_ indicate the times for systole and diastole, respectively; and SV denotes stroke volume (see e-Figure 1 in the online supplement). For each animal and stable experimental condition, one hundred beats from the pressure trace recordings were analyzed beat-by-beat with visual identification of the end-diastolic pressure and the dicrotic notch. According to the initial descriptions [15–18, 21], calibration constants were calculated with the first six to eight beats within a series, randomly chosen over respiratory cycles, i.e., calibration was performed for individual animals, in all experimental steps, for each of the four methods. We obtained the reference stroke volume for each respective beat by the integration of the systolic portion of the pulmonary artery flow from the ultrasound flow probe.

For immediate trend analysis, three randomly chosen respiratory cycles were chosen and the inspiratory heartbeat compared to the early and late expiratory heartbeats. The precision for each method expressed as the least significant change (i.e., the smallest true change detectable with 95% certainty) was calculated with data from expiratory beats only, as this represents the minimal physiological variation of stroke volume [23].

Pulmonary artery elastance (*E*_*a*_ = mPAP/SV) was calculated from the reference stroke volumes and the mean pulmonary artery pressure of the given beat as a surrogate of the end-systolic pressure [24].

### Statistical analysis

SigmaPlot 12.5 (Systat Software, Germany) and SPSS 25 (IBM Corporation, USA) were used for statistical and graphical analyses. Normal distribution was assessed with the Shapiro-Wilk test to decide between parametric or non-parametric tests. Comparisons between conditions were done with paired Student’s *t* test or Wilcoxon signed ranks test for the PEEP states and with one-way repeated measures analysis of variance (ANOVA) with post hoc Bonferroni correction or Friedman’s test with post hoc Tukey’s correction for the volume states (baseline, bleeding, and retransfusion). Comparisons between experimental conditions and methods were done with two-way repeated measures ANOVA (within-subject factors experimental condition and method). For the ANOVA, sphericity was assessed with Mauchly’s test and corrected for with the Greenhouse-Geisser procedure.

Data is given as mean ± standard deviation or median (range), depending on the normal distribution. Method agreement was assessed with the Bland-Altman analysis of bias (as an indication of accuracy) and precision (by the limits of agreement and the percentage error) [25] with correction for multiple repeated measurements [26] and Pearson’s correlation coefficients with linear regressions performed with the least square methods. Changes over time and trending were addressed using the 4-quadrant plot (with percentage concordance calculation [27] and Lin’s correlation coefficient [28]) and polar plots [29] by averaging one hundred beats per condition and comparing the inspiratory to the early and late expiratory beats. The zone of exclusion was chosen to be ± the least significant change, since a change lower than that would not be considered statistically relevant. Multiple linear regression for the computing constants and the method bias was done with standard hemodynamic parameters as predictors.

## Results

One animal died before any measurements were taken. The second animal developed ventricular fibrillation at the beginning of *bleeding* precluding further analysis [19]. Overall, 4300 individual beats were analyzed. Baseline hemodynamic data are given in Table 1. Baseline characteristics have been published before [19].

**Table 1:** Baseline hemodynamics per condition

	PEEP 5 cmH_2_O	PEEP 10 cmH_2_O	*p* value	Baseline	Bleeding	Retransfusion	*p* value
Heart rate [s^−1^]	88 ± 15	95 ± 23	0.25*	100 ± 20	128 ± 26	108 ± 22	< 0.001
Mean arterial pressure [mmHg]	62 ± 9	60 ± 13	0.378	58 ± 10	48 ± 10	63 ± 12	< 0.001
Mean pulmonary artery pressure [mmHg]	19 ± 3	19 ± 4	0.729	19 ± 2	17 ± 3	25 ± 4	< 0.001
Cardiac output [L/min]	2.94 ± 0.62	2.73 ± 0.67	0.084	2.82 ± 0.73	2.45 ± 0.62	3.40 ± 0.54	< 0.001
Stroke volume (mL)	34.8 ± 11.1	31 ± 12.5	< 0.001*	29.4 ± 11.1	20.6 ± 9.9	33.4 ± 10.9	< 0.001^†^

Part of these data has been previously published [19]

*Signed rank test

^†^Friedman’s ANOVA on ranks

### Method comparison

**Fig. 1:**
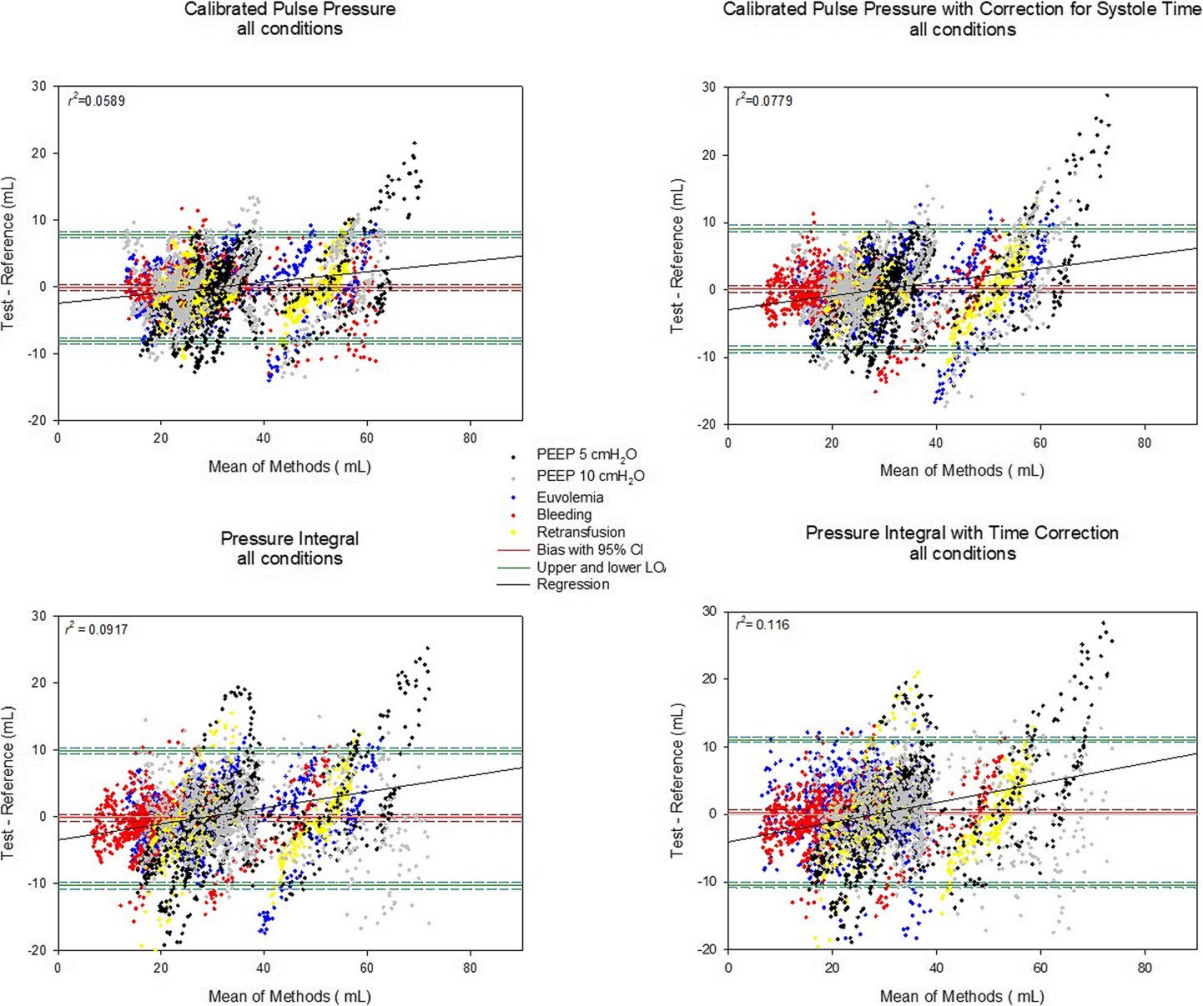
Bland-Altman plots for the four methods and all experimental steps. One hundred stroke volumes per animal and experimental condition are displayed, adding up to a total of 4300 beats. The experimental conditions are differentiated according to the color code in the legend (black cross, PEEP 5 cmH_2_O; grey cross, PEEP 10 cmH_2_O; blue cross, baseline; red cross, bleeding; yellow cross, retransfusion)

The average reference stroke volume for all conditions combined was 30 ± 12 mL. All pulse contour-based methods for the estimation of stroke volume from the PA pressure tracing had a minimal bias compared to the reference method, ranging from − 0.11 mL (95% CI − 0.55 to 0.33) to 0.32 mL (95% CI − 0.06 to 0.70). Similar biases could be shown for individual animals (e-figures 2, 3, 4, 5 in the online supplement). The limits of agreement ranged from − 8.0 to 7.8 mL for the calibrated pulse pressure to − 10.4 to 11.1 mL for the time-corrected pressure integral. The correlation between increases in stroke volume and bias were influenced by an outlying animal (Fig. 1). Multiple linear regression did not identify relevant predictors of the slight increase in bias with increasing stroke volume (Tables e1, e2, e3 and e4 in the online supplement).

The percentage error for stroke volumes varied from 26% for pulse pressure to 30% for time-corrected pulse pressure and 37% for the integration methods. Methods did not influence the bias within the PEEP levels (*p* = 0.214 for interaction) but showed an interaction with the volume state (*p* < 0.001, Tables e5 and e6 in the online supplement). Still, zero was included within the 95% confidence intervals of all biases (indicating that there was no significant difference in the bias to a value of zero). The calibration constants for each method did not significantly change between the PEEP levels, while they tended to change with the volume state, mainly in *retransfusion* (Table e7 in the electronic supplement). From the standard hemodynamic parameters, heart rate contributed most to predict changes in the constant in a multiple linear regression, even though at a low level of correlation (Tables e8, e9, e10 and e11 in the online supplement).

**Fig. 2:**
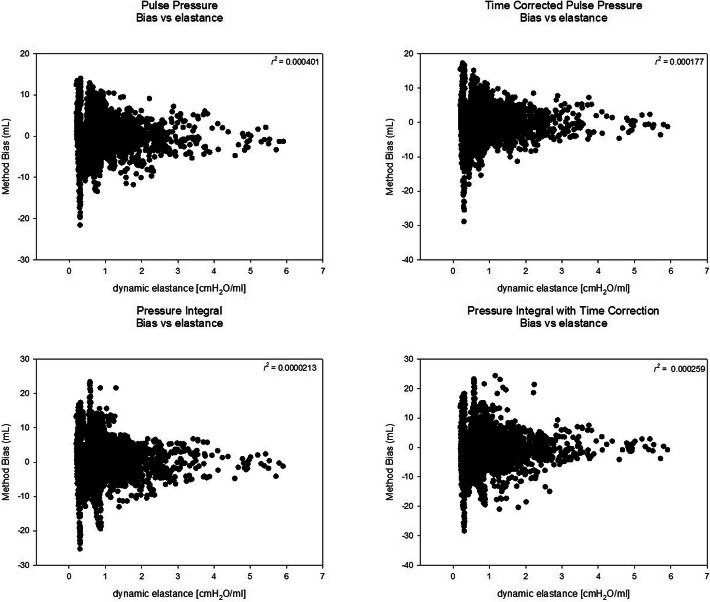
The relationship between dynamic elastance of the pulmonary artery and bias of the stroke volume estimate is shown. There is no correlation, but increasing precision with increasing elastance for all four methods investigated

The bias varied widely at low PA elastance values, but in both directions, independently of the method used. With increasing dynamic elastance, the precision increased. There is literally no correlation between elastance and bias (Fig. 2).

### Least significant change and interchangeability with the reference method

Our flow probe reference method had a least significant change (LSC) of 3.7 mL. The calibrated pulse pressure method had the lowest LSC of 2.7 mL. The time-corrected pulse pressure had a least significant change of 4.4 mL, and for the integration methods, it was 5 mL. Based on the average stroke volume of 30 mL, the LSC of the reference method translates into a precision of 12.3%. This is higher than what the manufacturer reports (10%).

As our percentage error is a combination of the error in the test method and the reference method, we have back-calculated the true percentage error of our methods, based on a reference method precision, as proposed by Montenij et al. [25] and Critchley and Critchley [30]. They are 24% and 27% for the pulse pressure methods and 35% for the integration methods.

### Trend analysis

**Fig. 3:**
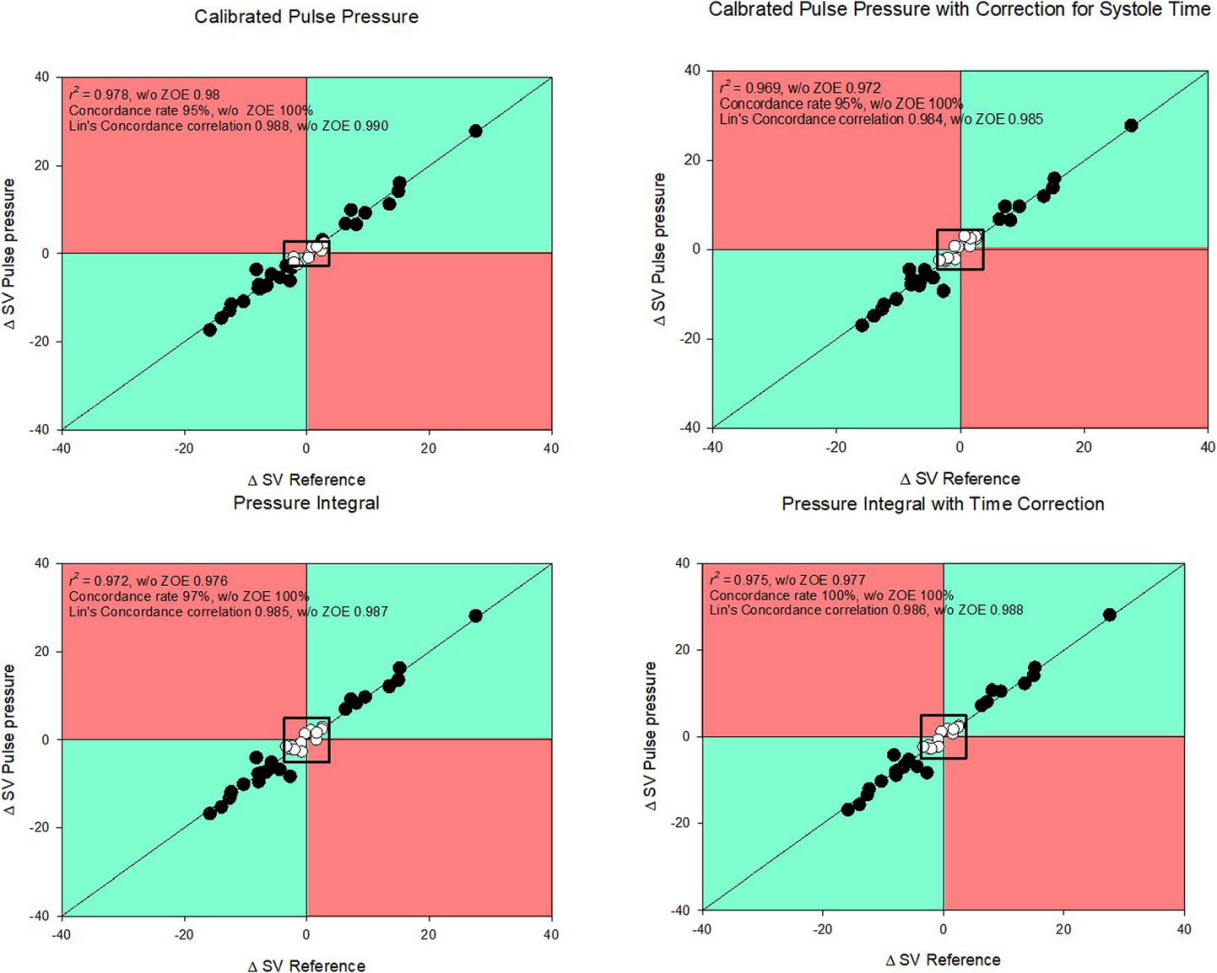
Four quadrant plots for trends in stroke volumes. One hundred stroke volumes per animal and condition were averaged, and the stepwise difference between the experimental conditions calculated. Thirty-four such comparisons could be made. The central zone of exclusion contains values within the range of the least significant change

**Fig. 4:**
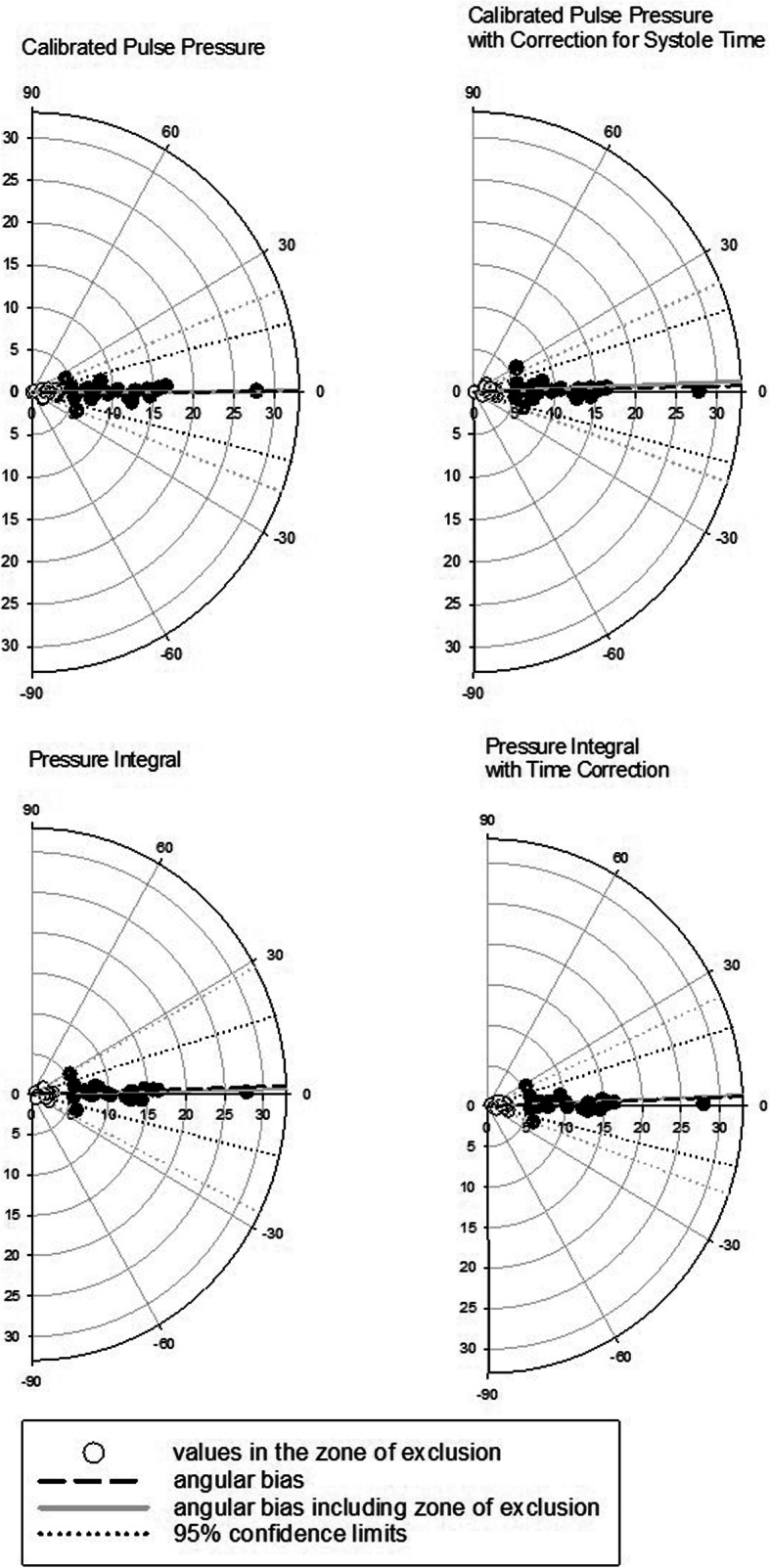
Polar plots for trending, with the same data as Fig. 3. The central zone of exclusion contains values within the range of the least significant change

The averaged stroke volumes over one hundred heartbeats trended changes in cardiac output for all investigated methods with high concordance rates (95 to 100%). Correlation coefficients were high with *r*^2^ from 0.969 to 0.978 and all values lay close to the line of identity. Lin’s concordance correlation coefficient indicated very high correlation [0.985 (0.98–0.988), Fig. 3]. The exclusion of a central exclusion zone (± LSC) improved all trending parameters (Fig. 3). The angular bias in the polar plots was below 3° for all methods (Fig. 4).

The Bland-Altman analysis for changes showed a minimal bias ranging from 0.03 to 0.06 mL. The limits of agreement increased from − 2.7 to 2.8 mL for the pulse pressure method and from − 3.2 to 3.1 mL for the methods based on integration. The resulting percentage error for changes for all methods was within a range of 12% of the average stroke volume (e-Figure 6).

**Fig. 5:**
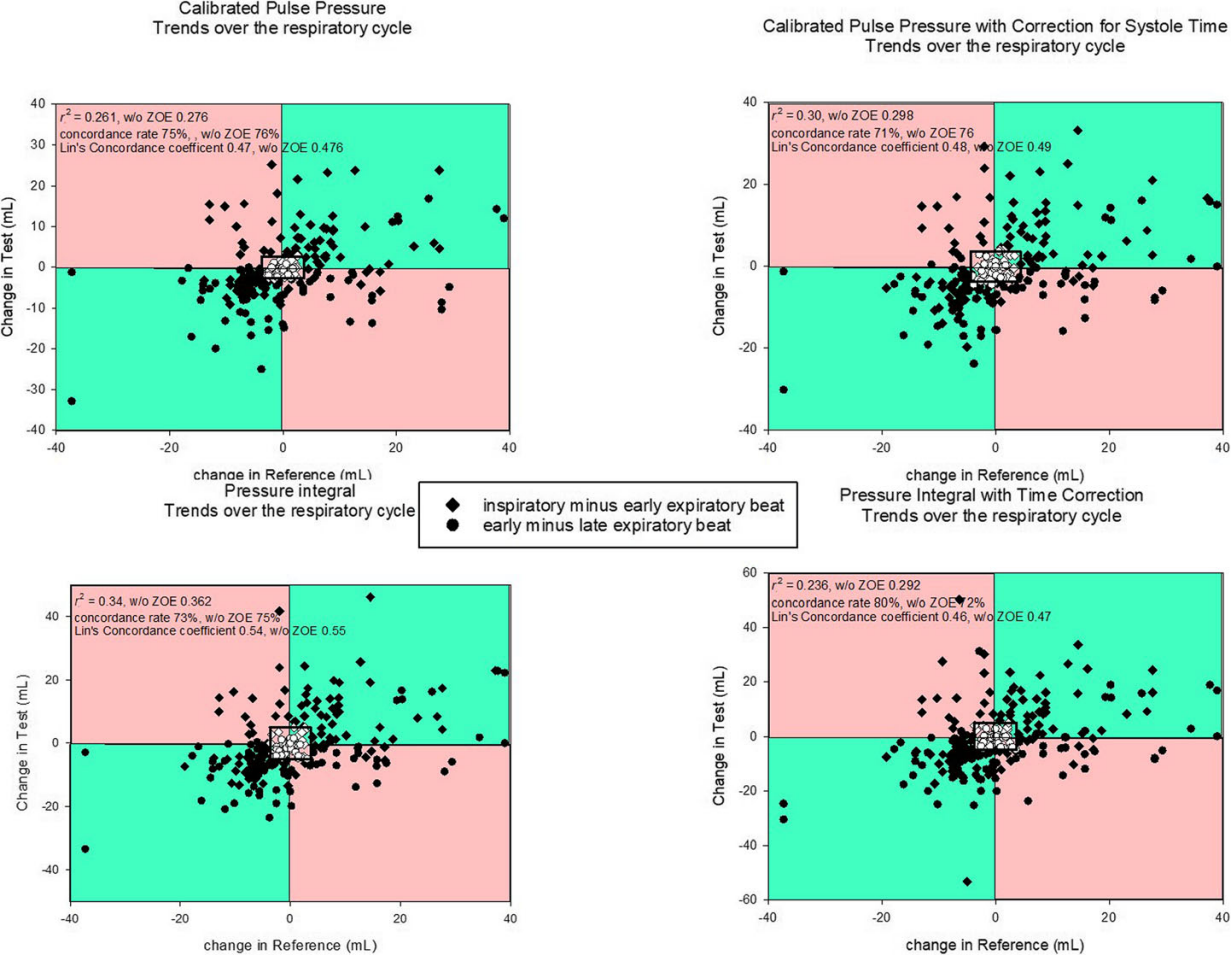
Beat-by-beat trending abilities over three respiratory cycles. The central zone of exclusion contains data within the range of the least significant change

For immediate beat-by-beat trending within the respiratory cycle, concordance rates between 71 and 80% was achieved with *r*^2^ from 0.23 to 0.34 (Fig. 5). Lin’s concordance correlation coefficient was moderate [0.475 (0.46–0.54)]. The Bland-Altman plot for changes (e-Figure 7) again indicates a small bias, but large limits of agreement.

## Discussion

Our study shows that simple methods based on pulse contour analysis of the pulmonary artery pressure trace are capable of estimating right ventricular stroke volume with a small, clinically insignificant bias and trend changes very well. The smallest bias was found for the simplest method, i.e., calibrated pulse pressure. The limits of agreement and the percentage error were at the border of clinical acceptance for stroke volumes [30], but the averaging of one hundred heartbeats rendered an almost perfect ability to trend changes in cardiac output. This could correct for the rather low precision of an individual stroke volume measurement, where the wide limits of agreement may render point measurements unreliable. Given the heart rate of the animals, this averaging would report fast changes within around 1 min, which is below the time resolution of any thermodilution system, be it intermittent or continuous. Since we also show that the beat-by-beat tracking is—even though acceptable—less favorable than averaging one hundred beats, the optimal running average or filtering rate would still need to be determined and might depend on the clinical scenario. Continuous thermodilution offers practical advantages and is less operator dependent, but also less accurate than intermittent thermodilution [31, 32] and limited by varying time delays of cardiac output results, which may render rapid clinical deteriorations undetectable [12, 33]. A method as ours, based on pulmonary pulse contour could help to circumvent these inherent limitations to the PAC. Systems for the determination of stroke volume variation with pulse contour based on transpulmonary thermodilution, i.e., left-sided or arterial pulse contour analysis, are limited by pulmonary factors. Respiratory rate, tidal volumes, and lung compliance may introduce an unpredictable phase shift in the stroke volume signals between the right and left ventricle. Right ventricular afterload increases may therefore be a reason for left-sided stroke volume variation, even though averaged right and left stroke volumes must equal each other [8]. Measurements upstream of the lung may not be exposed to these issues, since the right heart directly handles venous return and therefore changes in volume state [19, 20, 34]. Stroke volume variation may then be of particular interest. Since volume challenges were not part of the study protocol, this question cannot yet be answered. Before a reliable threshold for variations can be defined, the overall measurement performance of a method should be evaluated, which was the aim of this study.

In contrast to previous publications reporting similar strong correlations of estimated and measured stroke volumes [15–18], we assessed the methods based on bias and precision according to Bland and Altman [35, 36] and four-quadrant and polar plots and concordance [27]. Since we have repeated measurements, changes in the variance within the repeated measurements were accounted for [25, 26].

The proportionality between the pulse contour and stroke volume is represented in the calibration constant of the respective methods. Pressure and stroke volume in the pulmonary artery are the components of pulmonary elastance. PEEP may increase right ventricular afterload and pulmonary vascular impedance [7], but we observed no change in calibration constants with the change in PEEP. We found no correlation between the bias and the pulmonary artery elastance, but increasing precision with increasing elastance. This is in contrast to reports suggesting reduced accuracy of thermodilution measurements in pulmonary hypertension [32] and may, if verified in further studies, be a particular strength of our method in patients with acute or chronic pulmonary hypertension. Our calibration constants seemed to change with changing volume state, particularly with *retransfusion*. As a limitation of our retrospective design, the PEEP challenge was unidirectional and of limited intensity. It may introduce spectrum bias. A further limitation is the reporting of individual stroke volumes from a limited sample of animals. This may lead to an artificial narrowing of the limits of agreement. We have counterbalanced this effect by reporting every animal separately in the online supplement. Sample size calculation for the Bland-Altman analysis has been proposed [37]. This was not possible due to the retrospective study. Since we were particularly interested in trending stroke volume changes, there was no way around reporting individual stroke volumes. As a graphical interpretation model, BA plots may not heavily rely on statistical interferences [25].

Our frequent recalibration may have contributed to the solid trending results. The need for recalibration is an intrinsic problem for any pulse contour system available [3]. As a PAC always offers the possibility for thermodilution and continuous cardiac output measurement systems exist, a self-calibrating system is conceivable and may render short-term changes in stroke volume detectable based on a pulse contour method. The calibrations performed in each condition certainly improved the ability to track changes in SV. This is not currently possible in clinical practice. Combining RV pulse contour SV with recalibration using rapid thermodilution may provide such option and should be assessed in future studies. As we have only used volume changes to change cardiac output, the accuracy of the method may be different for changes in inotropic state or vasoconstriction, and it is an inherent limitation of our study that we do not have thermodilution measurements.

We have used a precise, highly invasive beat-to-beat reference method with a manufacturer-reported precision of ± 10%. We have found a precision of 12.3%. This reference probe was also used to calculate the calibration constants. It may therefore be criticized that pulmonary pulse contour may work less accurately when calibrated with a thermodilution method, and we may have a coupling between the reference method and the investigational method. Given our percentage errors of 26 to 37% for our methods under investigation and the reference precision of 12.3%, we cannot claim that our methods would be interchangeable with a highly invasive flow probe. For this to be true, the percentage error should lie below 15% [25, 30]. Clinical interchangeability to an invasive device like a flow probe is not possible anyway. Further studies with thermodilution as a calibration signal are therefore needed. Still, the strength of an intraindividual calibration of the pulse contour signal would also remain in this setting. In the original studies, dye dilution [17, 18], electromagnetic flow meters [16], and intermittent thermodilution with a PAC correlated well with pulse contour methods [15]. None of the original authors described how cardiac output for calibration was measured with respect to the respiratory cycle. This may clearly affect the cardiac output from any dilutional method [38], and the related swings in transpulmonary pressures are a major contributor to changes in right ventricular stroke volume [39]. We have chosen our beats for calibration randomly with respect to airway pressures. The pressure swings induced by mechanical ventilation may however account for the large limits of agreement that we observed. It is therefore of particular importance that the calibration is done with accountance of respiration. Our study is further limited by the small number of animals, the short observation period for trend analysis, and the highly controllable setup of an experimental lab. Still, our results are consistent over the different tested methods and the published literature.

## Conclusion

PA pulse contour seems to offer the possibility to detect short-term changes in stroke volume with good accuracy and moderate precision and accurately trend stroke volumes over time. Combined with the detection of waveform changes or pattern recognition, a self-calibrating system for clinical monitoring should be technically feasible.

## Supplementary information

**Additional file 1: e-Figure 1.** Schematic description of the pulmonary pressure trace (red) and pressure and time components that were used in the four different stroke volume calculations. T_s_ and T_d_ denote systolic and diastolic time of the cardiac cycle. $\underset{T_0}{\overset{T_e}{\int }} Pdt$ indicates the pressure integral over time from T_0_ (begin of systole) to T_e_ (end of systole), whereby Te minus T_0_ equals T_s_, the duration of systole.

**Additional file 2: e-Figures 2 to 5.** Bland-Altman plots for the four methods and all animals with respect to the experimental state. 100 stroke volumes per animal and experimental condition are displayed. The animals are differentiated with a symbol- and color code. The dependencies of bias from the mean of stroke volumes show median *r*^2^ of 0.73 (0.0 to 0.95) for the pulse pressure method, 0.73 (0.0 to 0.94) for the time corrected pulse pressure method, 0.8 (0.01 to 0.95) for the integration method and 0.84 (0.01 to 0.94) for the time corrected pressure integration method.

**Additional file 3: e-Figure 6.** Bland-Altman plots for changes over experimental conditions. The same data as in Fig. 3 in the main article were used.

**Additional file 4: e-Figure 7.** Bland-Altman plots for changes over the respiratory cycle. The same data as in Fig. 5 were used.

**Additional file 5.** Supplementary tables.

## Data Availability

The datasets used and/or analyzed during the current study are available from the corresponding author on reasonable request.

## Abbreviations

ANOVA: Analysis of variance
*E*_dyn_: Dynamic elastance
HR: Heart rate
*K*_*x*_: Calibration constant
LSC: Least significant change
MAP: Mean arterial pressure
PA: Pulmonary artery
PAC: Pulmonary artery catheter
PAP: Pulmonary artery pressure
*P*_ed_: End-diastolic pressure
PEEP: Positive end-expiratory pressure
PP: Pulse pressure
*P*_sys_: Systolic pressure
SV: Stroke volume
*T*_*e*_: End-systole time (dicrotic notch)
*T*_*d*_: Diastolic time
*T*_*s*_: Dystolic time
