# Supplementary figures and images for: Right ventricular stroke volume assessed by pulmonary artery pulse contour analysis
Source: Intensive Care Med Exp. 2020 Oct 7;8:58. doi: 10.1186/s40635-020-00347-7 (PMC7539259; doi:10.1186/s40635-020-00347-7)

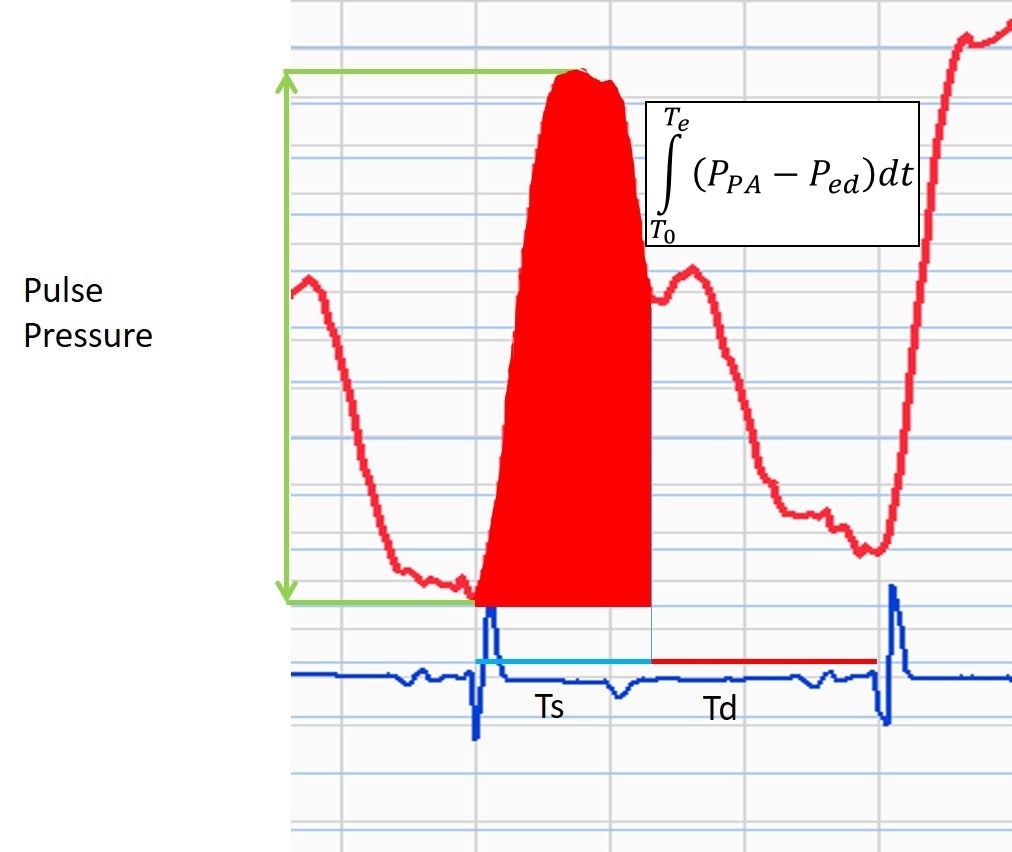

Supplement: Supplementary file 1 — Additional file 1: e-Figure 1. Schematic description of the pulmonary pressure trace (red) and pressure and time components that were used in the four different stroke volume calculations. Ts and Td denote systolic and diastolic time of the cardiac cycle. $\underset{T_0}{\overset{T_e}{\int }} Pdt$∫T0TePdt indicates the pressure integral over time from T0 (begin of systole) to Te (end of systole), whereby Te minus T0 equals Ts, the duration of systole. [file 40635_2020_347_MOESM1_ESM.jpg]

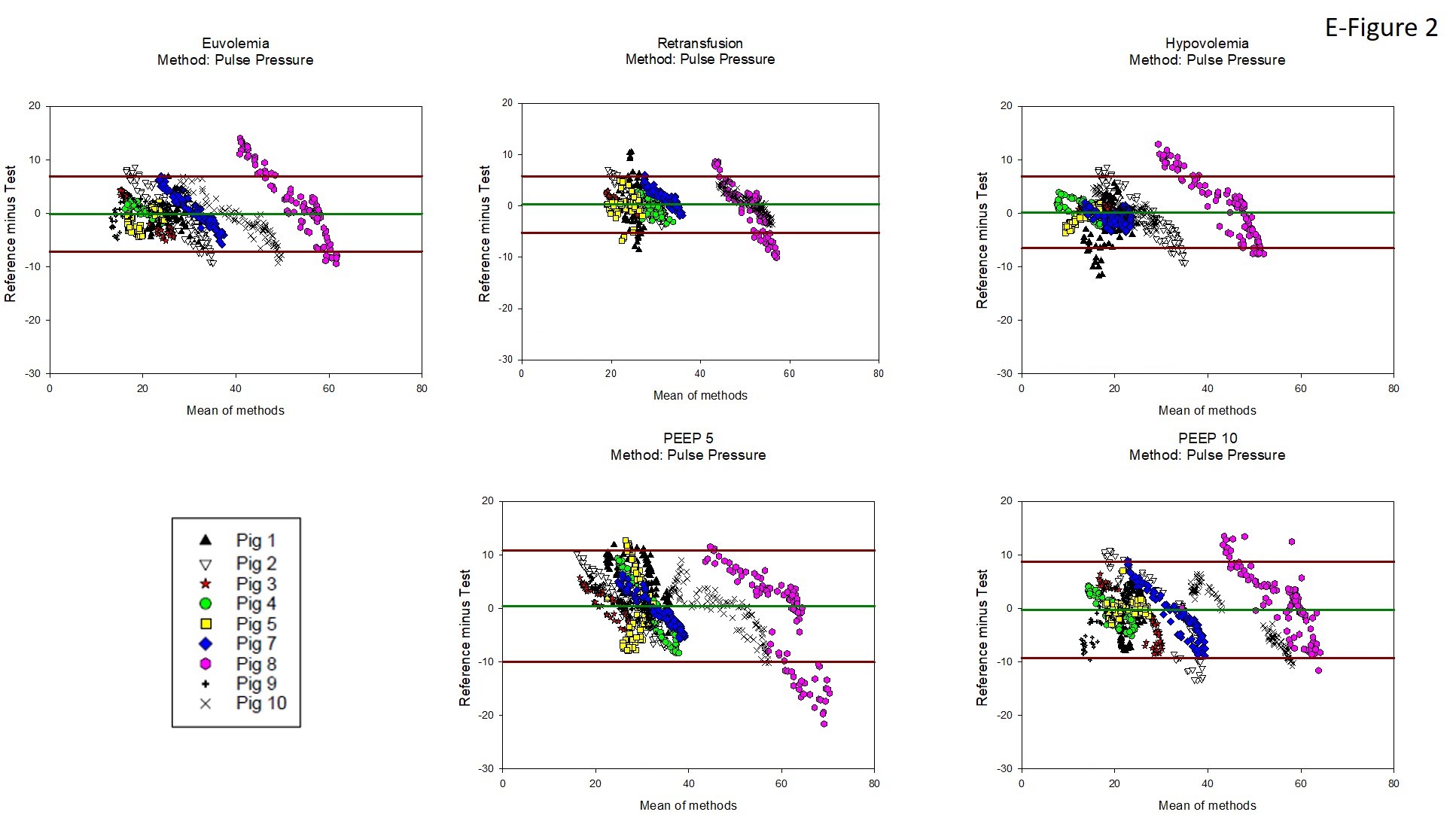

Supplement: Supplementary file 2 — Additional file 2: e-Figures 2 to 5. Bland-Altman plots for the four methods and all animals with respect to the experimental state. 100 stroke volumes per animal and experimental condition are displayed. The animals are differentiated with a symbol- and color code. The dependencies of bias from the mean of stroke volumes show median r2 of 0.73 (0.0 to 0.95) for the pulse pressure method, 0.73 (0.0 to 0.94) for the time corrected pulse pressure method, 0.8 (0.01 to 0.95) for the integration method and 0.84 (0.01 to 0.94) for the time corrected pressure integration method. [file 40635_2020_347_MOESM2_ESM.zip › e-figure2.jpg]

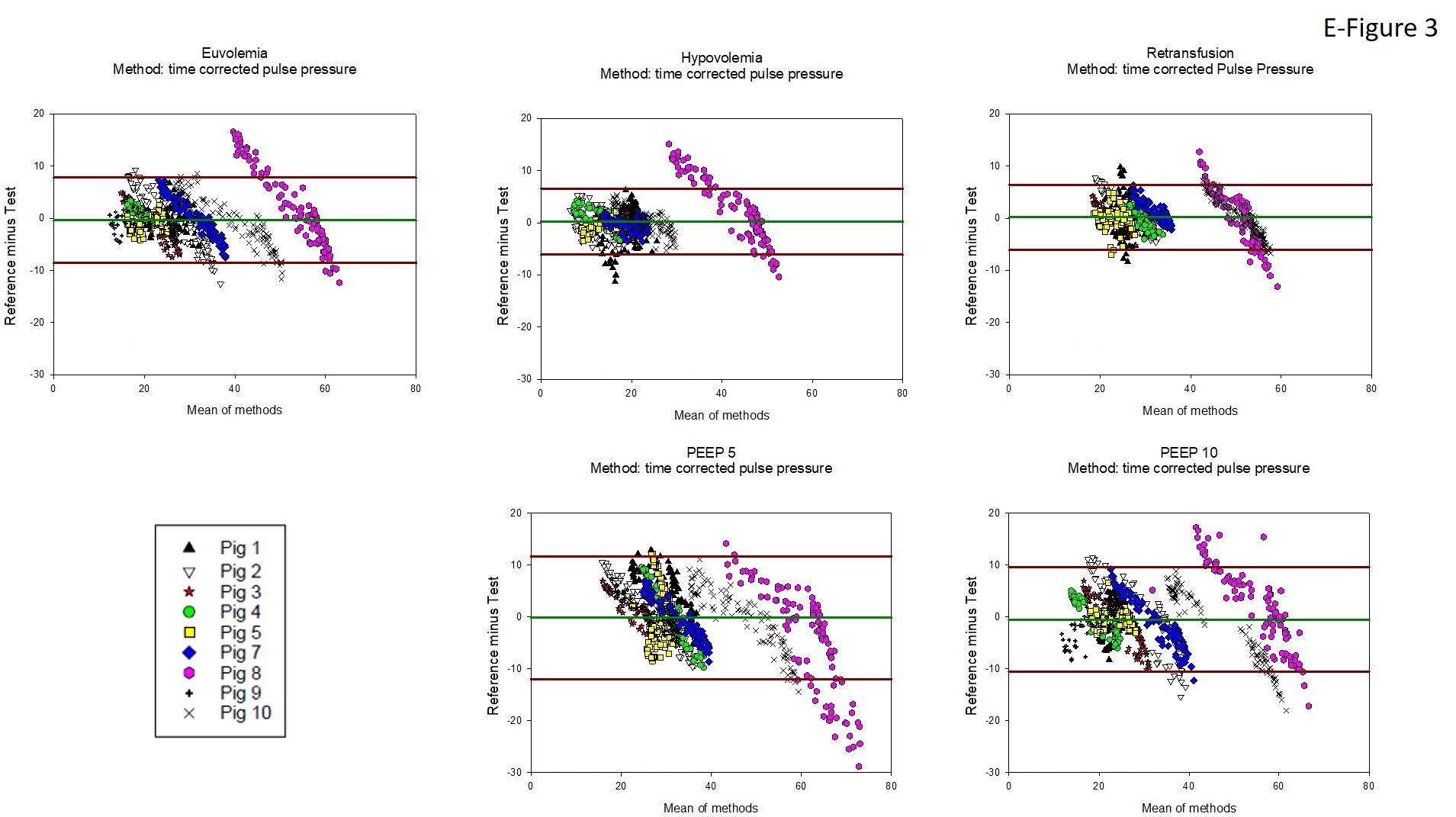

Supplement: Supplementary file 2 — Additional file 2: e-Figures 2 to 5. Bland-Altman plots for the four methods and all animals with respect to the experimental state. 100 stroke volumes per animal and experimental condition are displayed. The animals are differentiated with a symbol- and color code. The dependencies of bias from the mean of stroke volumes show median r2 of 0.73 (0.0 to 0.95) for the pulse pressure method, 0.73 (0.0 to 0.94) for the time corrected pulse pressure method, 0.8 (0.01 to 0.95) for the integration method and 0.84 (0.01 to 0.94) for the time corrected pressure integration method. [file 40635_2020_347_MOESM2_ESM.zip › e-figure3.jpg]

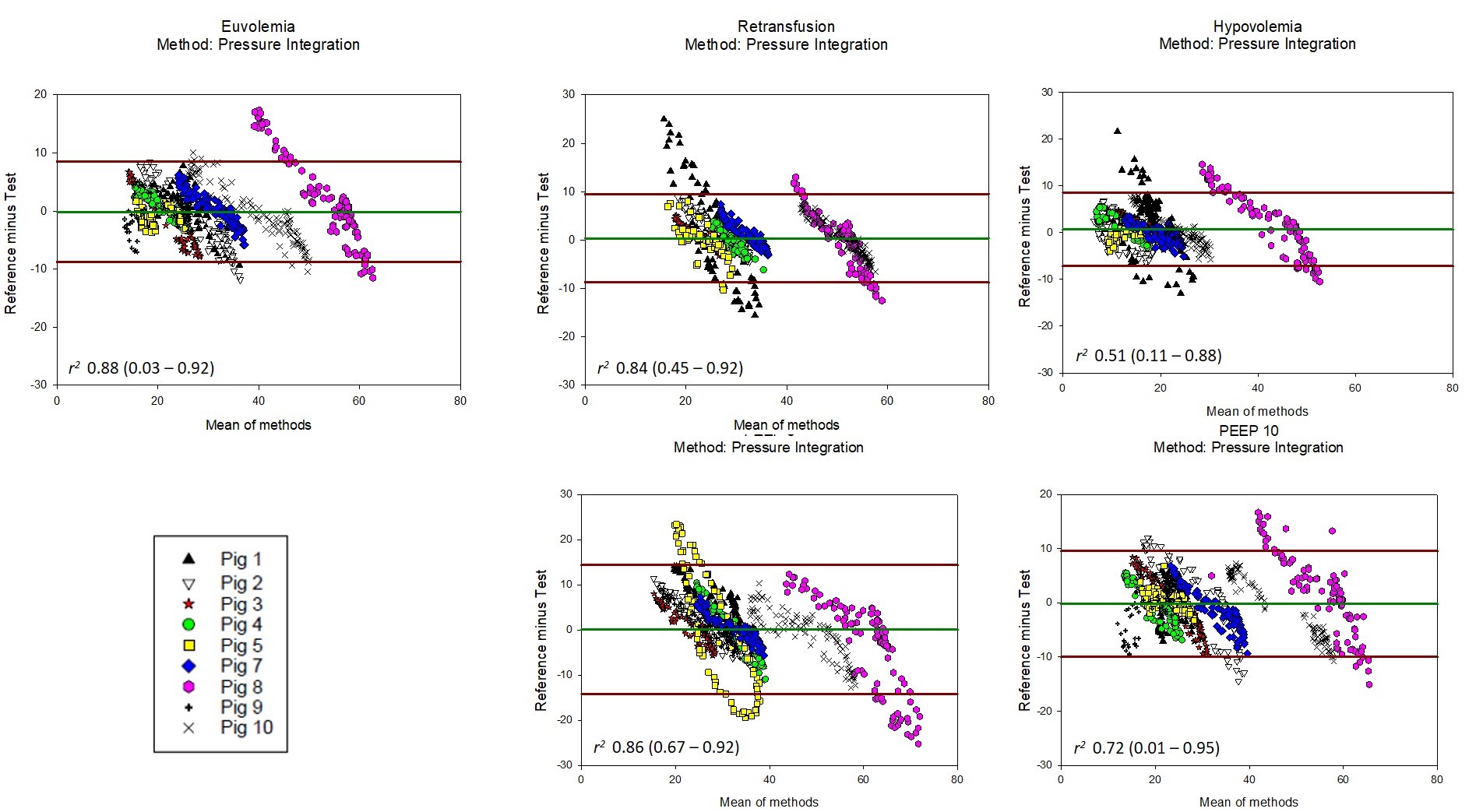

Supplement: Supplementary file 2 — Additional file 2: e-Figures 2 to 5. Bland-Altman plots for the four methods and all animals with respect to the experimental state. 100 stroke volumes per animal and experimental condition are displayed. The animals are differentiated with a symbol- and color code. The dependencies of bias from the mean of stroke volumes show median r2 of 0.73 (0.0 to 0.95) for the pulse pressure method, 0.73 (0.0 to 0.94) for the time corrected pulse pressure method, 0.8 (0.01 to 0.95) for the integration method and 0.84 (0.01 to 0.94) for the time corrected pressure integration method. [file 40635_2020_347_MOESM2_ESM.zip › e-figure4.jpg]

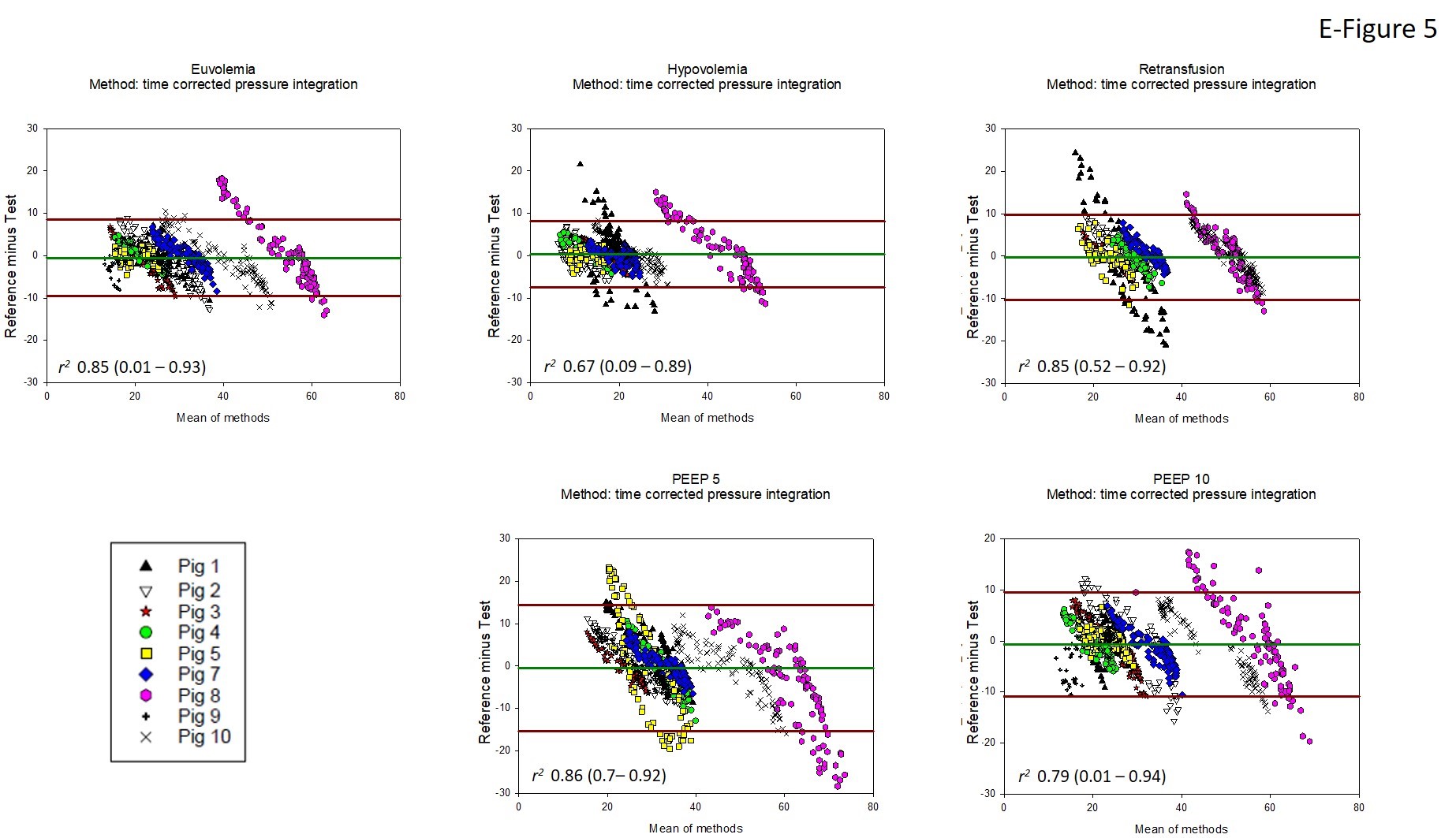

Supplement: Supplementary file 2 — Additional file 2: e-Figures 2 to 5. Bland-Altman plots for the four methods and all animals with respect to the experimental state. 100 stroke volumes per animal and experimental condition are displayed. The animals are differentiated with a symbol- and color code. The dependencies of bias from the mean of stroke volumes show median r2 of 0.73 (0.0 to 0.95) for the pulse pressure method, 0.73 (0.0 to 0.94) for the time corrected pulse pressure method, 0.8 (0.01 to 0.95) for the integration method and 0.84 (0.01 to 0.94) for the time corrected pressure integration method. [file 40635_2020_347_MOESM2_ESM.zip › e-figure5.jpg]

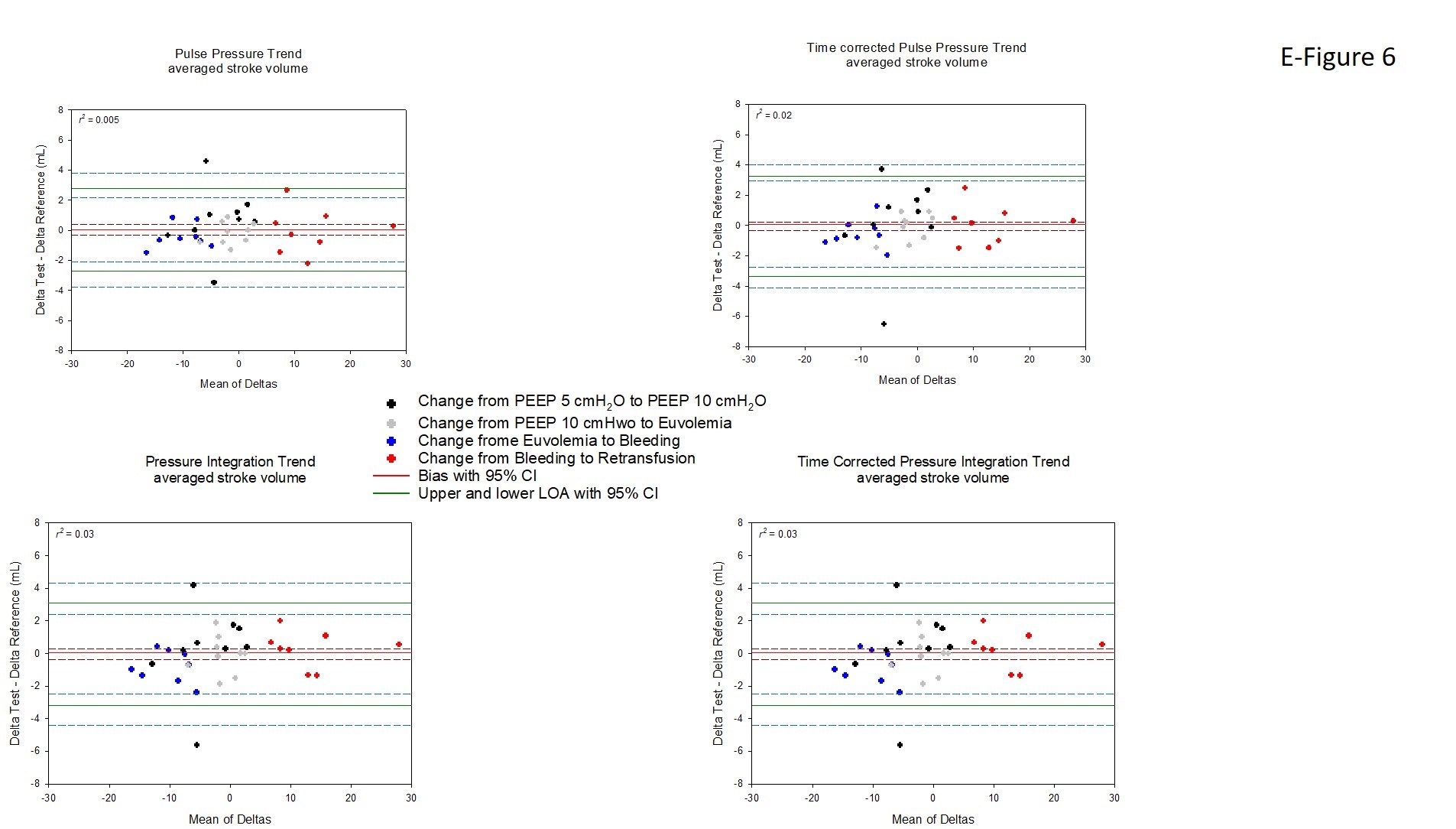

Supplement: Supplementary file 3 — Additional file 3: e-Figure 6. Bland-Altman plots for changes over experimental conditions. The same data as in Fig. 3 in the main article were used. [file 40635_2020_347_MOESM3_ESM.jpg]

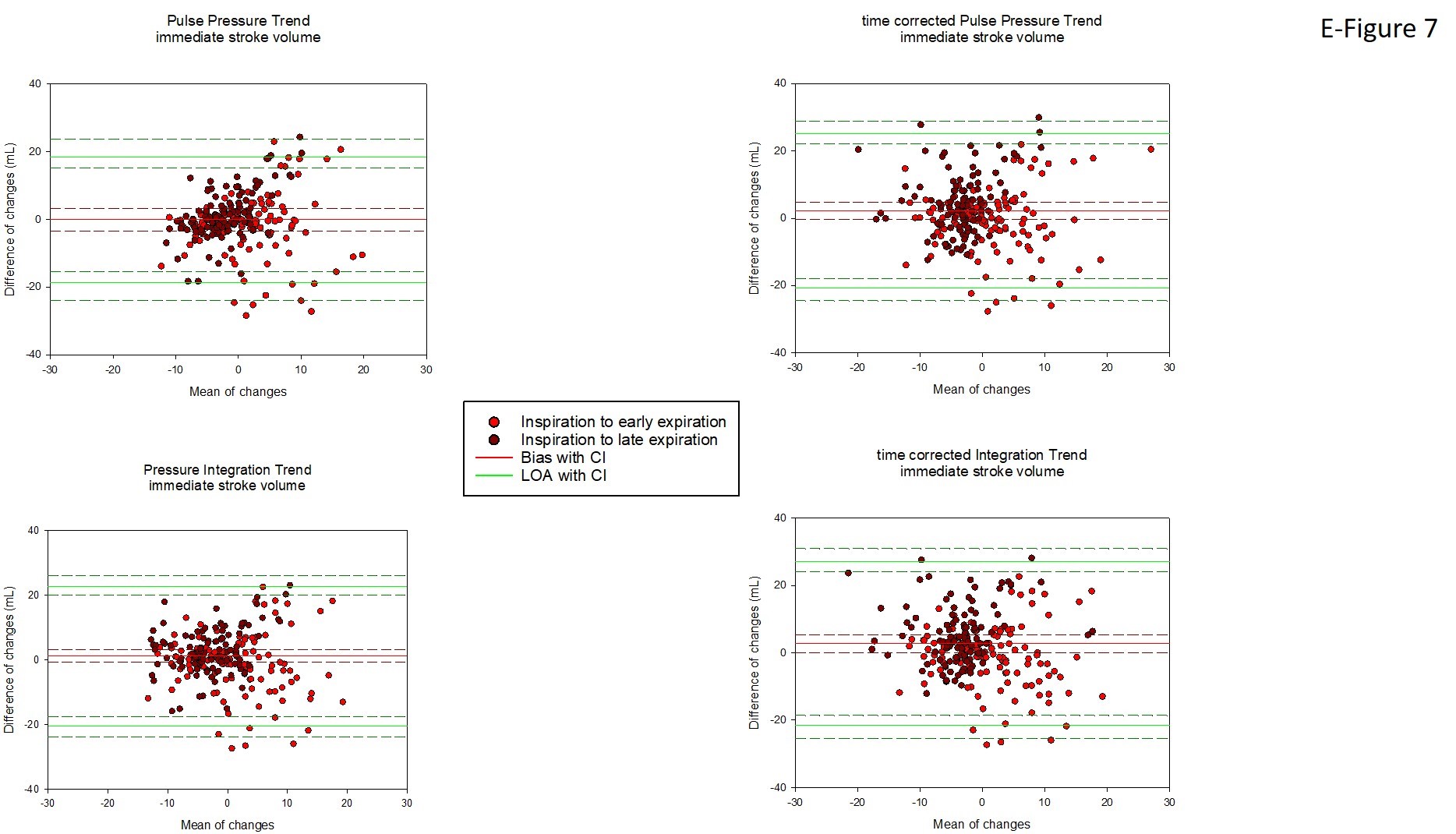

Supplement: Supplementary file 4 — Additional file 4: e-Figure 7. Bland-Altman plots for changes over the respiratory cycle. The same data as in Fig. 5 were used. [file 40635_2020_347_MOESM4_ESM.jpg]
