# Supplementary material for: Right ventricular stroke volume assessed by pulmonary artery pulse contour analysis
Source: Intensive Care Med Exp. 2020 Oct 7;8:58. doi: 10.1186/s40635-020-00347-7 (PMC7539259; doi:10.1186/s40635-020-00347-7)
Supplement: Supplementary file 5 — Additional file 5. Supplementary tables. [file 40635_2020_347_MOESM5_ESM.docx]

|  | | |  | |  | | |  | |  |
| --- | --- | --- | --- | --- | --- | --- | --- | --- | --- | --- |
|  | | |  | |  | | |  | |  |
| **Multiple linear regression model for the pulse pressure method** | | | | | | | | |  |  |
|  | non standardized coefficients | | | standardized coefficients | | p-value |  | |  |  |
|  | B | 95% CI | | Beta | |  |  | |  |  |
| (constant) | -1.351 | -2.627 to -.0.75 | |  | | 0.038 |  | |  |  |
| MAP | 0.008 | -0.002 to 0.019 | | 0.025 | | 0.120 |  | |  |  |
| mPAP | 0.084 | 0.052 to 0.117 | | 0.089 | | 0.000 |  | |  |  |
| HeartRate | -0.009 | -0.015 to -0.003 | | -0.054 | | 0.006 |  | |  |  |
| Elastance | -0.080 | -.0297 to 0.137 | | -0.015 | | 0.469 |  | |  |  |
| **Table e1:** Dependent Variable: Bias, R2= 0.012, DeltaR2 = 0.012, p< 0.001 | | | | | | | | |  |  |
|  |  |  | |  | |  |  | |  |  |
|  |  |  | |  | |  |  | |  |  |
| **Multiple linear regression model for the time corrected pulse pressure method** | | | | | | | | | | |
|  | non standardized coefficients | | | standardized coefficients | | p-value |  | |  |  |
|  | B | 95% CI | | Beta | |  |  | |  |  |
| (Constant) | -1.281 | -2.718 to 0.156 | |  | | 0.081 |  | |  |  |
| MAP | 0.017 | 0.005 to 0.029 | | 0.046 | | 0.004 |  | |  |  |
| mPAP | 0.081 | 0.004 to 0.117 | | 0.076 | | <0.001 |  | |  |  |
| HeartRate | -0.014 | -0.021 to -0.007 | | -0.074 | | <0.001 |  | |  |  |
| Elastance | 0.063 | -0.181 to 0.308 | | 0.011 | | 0.612 |  | |  |  |
| **Table e2:** Dependent Variable: Bias, R2= 0.014, DeltaR2 = 0.014, p< 0.001 | | | | | | | | |  |  |
|  |  |  | |  | |  |  | |  |  |
|  |  |  | |  | |  |  | |  |  |
| **Multiple linear regression model for the pressure integration method** | | | | | | | | | | |
|  | non standardized coefficients | | | standardized coefficients | | p-value |  | |  |  |
|  | B | 95% CI | | Beta | |  |  | |  |  |
| (Constant) | -2.860 | -4.596 to -1.124 | |  | | 0.001 |  | |  |  |
| MAP | -0.004 | -0.018 to 0.011 | | -0.008 | | 0.601 |  | |  |  |
| mPAP | 0.112 | 0.068 to 0.156 | | 0.087 | | 0.000 |  | |  |  |
| HeartRate | 0.006 | -0.003 to 0.015 | | 0.027 | | 0.167 |  | |  |  |
| Elastance | 0.275 | -0.021 to 0.570 | | 0.038 | | 0.068 |  | |  |  |
| **Table e3:** Dependent Variable: Bias, R2= 0.006, DeltaR2 = 0.006, p< 0.001 | | | | | | | | |  |  |
|  |  |  | |  | |  |  | |  |  |
| **Multiple linear regression model for the time corrected pressure integration method** | | | | | | | | | | |
|  | non standardized coefficients | | | standardized coefficients | | p-value |  | |  |  |
|  | B | 95% CI | | Beta | |  |  | |  |  |
| (Constant) | -1.078 | -2.692 to 0.536 | |  | | 0.19 |  | |  |  |
| MAP | -0.001 | -0.014 to 0.013 | | -0.001 | | 0.927 |  | |  |  |
| mPAP | 0.138 | 0.097 to 0.179 | | 0.114 | | 0 |  | |  |  |
| HeartRate | -0.021 | -0.029 to -0.013 | | -0.099 | | 0 |  | |  |  |
| Elastance | 0.124 | -0.15 to 0.399 | | 0.018 | | 0.375 |  | |  |  |
| **Table e4:** Dependent Variable: Bias, R2= 0.023, DeltaR2 = 0.023, p< 0.001 | | | | | | |  | |  |  |

|  |  |  | Limits of agreement | |  |  |
| --- | --- | --- | --- | --- | --- | --- |
| **PEEP 5 cm H_2_O** | r^2^ | Bias | lower | upper | SDDIFF ± SE |  |
| Pulse Pressure | 0.85 | -0.42 (-1.89 to 1.06) | -10.95 (-13.06 to -9.58) | 10.1 (8.75 to 12.23) | 5.37 ± 0.20 |  |
| time corrected pulse pressure | 0.84 | 0.15 (-1.63 to 1.94) | -11.83 (-14.48 to -10.17) | 12.14 (10.48 to 14.79) | 6.12 ± 0.25 |  |
| pressure integral | 0.77 | -0.10 (-1.84 to 1.64) | -14.48 (-16.83 to -12.85) | 14.28 (12.64 to 16.63) | 7.34 ± 0.24 |  |
| time corrected pressure integral | 0.76 | 0.44 (-1.32 to 2.22) | -14.48 (-16.85 to -12.82) | 15.38 (13.71 to 17.75) | 7.62 ± 0.24 |  |
| **PEEP 10 cm H_2_O** |  |  |  |  |  |  |
| Pulse Pressure | 0.89 | 0.27 (-0.44 to 0.99) | -8.76 (-9.63 to -8.03) | 9.3 (8.58 to 10.18) | 4.61 ± 0.11 |  |
| time corrected pulse pressure | 0.86 | 0.46 (-0.52 to 1.45) | -9.65 (-10.89 to -8.70) | 10.58 (9.63 to 11.81) | 5.16 ± 0.14 |  |
| pressure integral | 0.87 | 0.21 (-0.59 to 1.01) | -9.59 (-10.57 to -8.78) | 10.02 (9.21 to 11.00) | 5.00 ±0.13 |  |
| time corrected pressure integral | 0.86 | 0.64 (0.09 to 1.19) | -9.56 (-10.32 to -8.95) | 10.87 (10.22 to 11.59) | 5.22 ± 0.12 |  |
| **Table e5:** r^2^ for *stroke volume* with test method vs. reference method | | | |  |  |  |
| The bias is stable between PEEP levels (p=0.132) but varies with the method (p<0.001, no PEEP*method interaction, p=0.214) | | | | | |  |

|  |  |  |  |  |  |  |
| --- | --- | --- | --- | --- | --- | --- |
|  |  |  | Limits of agreement | |  |  |
| **Baseline** | r^2^ | Bias | lower | upper | SDDIFF ± SE |  |
| Pulse Pressure | 0.91 | 0.09 (-0.51 to 0.70) | -6.94 (-7.69 to -6.34) | 7.13 (6.53 to 7.88) | 3.59 ± 0.09 |  |
| time corrected pulse pressure | 0.88 | 0.38 (-0.42 to 1.18) | -7.80 (-8.82 to -7.03) | 8.57 (7.79 to 9.58) | 4.18 ± 0.11 |  |
| pressure integral | 0.87 | 0.12 (-0.78 to 1.01) | -8.51 (-9.65 to -7.66) | 8.75 (7.90 to 9.89) | 4.40 ± 0.12 |  |
| time corrected pressure integral | 0.86 | 0.48 (-0.36 to 1.32) | -8.54 (-9.59 to -7.83) | 9.51 (8.69 to 10.55) | 4.61 ± 0.12 |  |
| **Hypovolemia** |  |  |  |  |  |  |
| Pulse Pressure | 0.91 | -0.26 (-1.07 to 0.55) | -6.29(-7.43 to -5.55) | 5.77 (5.03 to 6.91) | 3.08 ± 0.11 |  |
| time corrected pulse pressure | 0.90 | -0.25 (-1.17 to 0.67) | -6.64 (-7.97 to -5.81) | 6.14 (5.31 to 7.47) | 3.26 ± 0.12 |  |
| pressure integral | 0.86 | -0.66 (-0.15 to 0.16) | -8.54 (-9.59 to -7.76) | 7.21 (6.43 to 8.26) | 4.02 ± 0.11 |  |
| time corrected pressure integral | 0.87 | -0.34 (-0.80 to 0.12) | -8.13 (-8.73 to -7.61) | 7.46 (6.93 to 8.05) | 3.98 ± 0.10 |  |
| **Retransfusion** |  |  |  |  |  |  |
| Pulse Pressure | 0.94 | -0.29 (-0.81 to 0.24) | -5.83 (-6.49 to -5.33) | 5.26 (4.76 to 5.92) | 2.83 ± 0.07 |  |
| time corrected pulse pressure | 0.92 | -0.20 (-0.58 to 0.18) | -6.46 (-6.95 to -6.03) | 6.06 (5.63 to 6.55) | 3.19 ± 0.08 |  |
| pressure integral | 0.86 | -0.38 (-1.02 to 0.26) | -9.53 (-10.33 to -8.86) | 8.77 (8.09 to 9.56) | 4.67 ± 0.12 |  |
| time corrected pressure integral | 0.82 | 0.29 (-0.39 to 0.97) | -9.83 (-10.68 to -9.09) | 10.41 (9.67 to 11.26) | 5.16 ± 0.13 |  |
| **Table e6:** r^2^ for *stroke volume* with test method vs. Stroke volume with the reference method | | | |  |  |  |
| The bias depends on the volume state (p=0.007) and varies with the method (p<0.001, PEEP*method interaction, p=0.001) | | | | | |  |

| **Calibration constants** | PEEP 5 cmH2O | PEEP 10 cmH2O | p-value | Baseline | Bleeding | Retransfusion | p-value |
| --- | --- | --- | --- | --- | --- | --- | --- |
| Pulse Pressure [mL*mmHg-1] | 3.42 ± 1.1 | 3.32 ± 1.1 | 0.455 | 3.21 ± 1.33 | 2.73 ± 1.44 | 2.31 ± 0.91 | 0.028 |
|  |  |  |  |  |  |  |  |
| time corrected Pulse Pressure [mL*mmHg-1*100 sec-1] | 0.101 ± 0.002 | 0.102 ± 0.003 | 0.83 | 0.099 ± 0.03 | 0.095 ± 0.045 | 0.074 ± 0.016 | 0.056 |
|  |  |  |  |  |  |  |  |
| Pressure Integral [mL*mmHg-1*sec-1] | 16.4 ± 3.7 | 16.6 ± 3.9 | 0.665 | 14.5 (13.0 - 26.8) | 14.1 (10.2 – 33.4) | 11.6 (9.7 - 18.0) | 0.151* |
| time corrected pressure integral [mL*mmHg-1*sec-1] | 8.6 ± 2.3 | 8.4 ± 2.4 | 0.613 | 8.28 ± 2.92 | 6.84 ± 3.01 | 5.97 ±1.23 | 0.001 |
| **Table e7:** * ANOVA on ranks, data as median (range) | |  |  |  |  |  |  |

**Multiple linear regression models for the calculation constants**

**Multiple linear regression model for the pulse pressure method**

|  | non standardized coefficients | | standardized coefficients |  |
| --- | --- | --- | --- | --- |
|  | B | 95% CI | Beta | p-value |
| (Constant) | 8.999 | 6.162 to 11.836 |  | 0.000 |
| MAP | -0.027 | -0.053 to 0.000 | -0.258 | 0.048 |
| PAP | -0.062 | -0.133 to 0.010 | -0.207 | 0.088 |
| CO | 7.176E-05 | 0.000 to 0.000 | 0.041 | 0.725 |
| HR | -0.033 | -0.046 to -0.021 | -0.681 | 0.000 |
| **Table e8:** Dependent Variable: pulse pressure constant, r^2^ 0.528, Delta r^2^ 0.528, p <0.001 | | | | |

**Multiple linear regression model for the time corrected pulse pressure method**

|  |  |  |  |  |  |
| --- | --- | --- | --- | --- | --- |
|  | non standardized coefficients | | standardized coefficients | p-value |  |
|  | B | 95% CI | Beta |  |  |
| (Constant) | 0.241 | 0.150 to 0.331 |  | 0.000 |  |
| MAP | -0.001 | -0.002 to 0.000 | -0.280 | 0.086 |  |
| PAP | -0.001 | -0.003 to 0.002 | -0.084 | 0.578 |  |
| CO | -9.268E-06 | 0.000 to 0.000 | -0.210 | 0.160 |  |
| HR | -0.001 | -0.001 to 0.000 | -0.505 | 0.003 |  |
| **Table e9:** Dependent Variable: time corrected pulse pressure constant, r^2^ 0.251, Delta r^2^ 0.251, p 0.024 | | | | | |

**Multiple linear regression model for the integration method**

|  | non standardized coefficients | | standardized coefficients | p-value |  |
| --- | --- | --- | --- | --- | --- |
|  | B | 95% CI | Beta |  |  |
| (Konstante) | 35.521 | 20.501 to 50.541 |  | 0.000 |  |
| MAP | -0.121 | -0.261 to 0.019 | -0.290 | 0.089 |  |
| PAP | -0.065 | -0.443 to 0.313 | -0.055 | 0.729 |  |
| CO | -0.001 | -0.003 to 0.001 | -0.158 | 0.311 |  |
| HR | -0.081 | -0.147 to -0.015 | -0.413 | 0.018 |  |
| **Table e10:** Dependent Variable: integration constant, r^2^ 0.178, Delta r^2^ 0.178, p 0.105 | | | | | |

**Multiple linear regression model for time corrected integration method**

|  | |  | |  |  |  |
| --- | --- | --- | --- | --- | --- | --- |
|  | non standardized coefficients | | | | standardized coefficients | p-value |
|  | B | | 95% CI | | Beta |  |
| (Konstante) | 19.161 | | 12.0 to 26.323 | |  | 0.000 |
| MAP | -0.041 | | -0.108 to 0.026 | | -0.184 | 0.224 |
| PAP | 0.027 | | -0.153 to 0.207 | | 0.043 | 0.763 |
| CO | -0.001 | | -0.002 to 0.000 | | -0.260 | 0.066 |
| HR | -0.067 | | -0.098 to -0.035 | | -0.644 | 0.000 |
| **Table e11:** Dependent Variable: integration constant, r^2^ 0.338, Delta r^2^ 0.338, p 0.003 | | | | | | |
